# Supplementary material for: Effects of building resilience skills among undergraduate medical students in a multi-cultural, multi-ethnic setting in the United Arab Emirates: A convergent mixed methods study
Source: PLoS One. 2025 Feb 27;20(2):e0308774. doi: 10.1371/journal.pone.0308774 (PMC11867382; doi:10.1371/journal.pone.0308774)
Supplement: S1 File — (DOCX) [file pone.0308774.s001.docx]

**Focus Group Protocol- Resilience Skills Course**

**Phase III Personal Journey (thus far)**

Reflect upon your personal journey since the beginning of Phase III (10 minutes)

- Thoughts, emotions, and behaviors, and their interplay
- Facilitators
- Stressors
- Challenges
- Coping mechanisms

How do you describe your personal evolution since the beginning of Phase III? (10 minutes)

- Thoughts, emotions, and behaviors
- Coping mechanisms
- Main influencing factors

Reflect upon the Resilience Skills Course, and how you perceive it affected you (15 minutes)

- Knowledge, skills, and competencies acquired
- Health and wellbeing
- Personal life
- Studies
- Coping mechanism
- Resilience

How would you describe the Resilience Skills Course (the overall experience)? (15 minutes)

- Strengths
- Opportunities for improvement
- Before/ after COVID-19
