# Supplementary material for: Effects of building resilience skills among undergraduate medical students in a multi-cultural, multi-ethnic setting in the United Arab Emirates: A convergent mixed methods study
Source: PLoS One. 2025 Feb 27;20(2):e0308774. doi: 10.1371/journal.pone.0308774 (PMC11867382; doi:10.1371/journal.pone.0308774)
Supplement: S2 File — (DOCX) [file pone.0308774.s002.docx]

**Complete Output of Analysis**

| **Theme** | **Category** | **Exemplars** |
| --- | --- | --- |
| **Transition** | **Change** | 1M: “…it was really new…we were always in lectures in classrooms and then we had to put ourselves out there…”  2F: “…first day of Phase III compared to where I stand now, I think there has been a big change not only academically, as in increase in knowledge, but also personally… I learned and grew so much through the clinical experiences in terms of my personality… It is no longer about ‘a grade’ or how you performed in an exam…”  3M: “…COVID-19 was definitely something new, unpredictable. I felt stressed because we did not know how to react to the changes accompanying the pandemic; we did not know what to expect… I did not know what to expect from the online learning experience and how, in particular, we will compensate for not going to the hospital…I was looking forward to my surgery rotation, a lot of people told me that it is really nice that you are going to have so much fun, and then, when the COVID-19 happened, we could not go and attend the rotation… the biggest impact on me was the disappointment because I really wanted to experience the rotation. Luckily, we have surgery next year…”  4F: “…for me, it was just the difference of ‘how to learn’. I was so used to having the material in front of me… When you are in the hospital, you sort of need to seek that information on your own whether that is asking doctors, getting laboratory results, uh being in the Operating Theatre for like a surgery, for example, so you need to be more active in your learning, to go out of your way to learn. That was the biggest shift for me…” |
|  | **Challenges** | 1M: “…we had to … see and interact with patients. Doctors would actually ask us questions, and sometimes put us ‘on the spot’…”  2F: “…you find yourself right in front of the doctor, you get direct feedback, then and there…”  3M: “…COVID-19 would count as a turning point which made our journey more challenging…”  5F: “…I think I can speak on behalf of everyone when I say this year has been challenging on many levels…the change of assessment from preclinical to clinical brought me a lot of stress. I consider it ‘unnecessary stress’. The stress was not because I did not perform well but it was because of the way the variation in the way the assessors would approach the assessment form. I understand every doctor has a different style, as they graduate from different universities and have different training experiences, but when it comes to assessments, one would assume that it is standard across all rotations and all the doctors. This was not the case for me…”  6F: “…a lot of the students do not know how to benefit from their time in the hospital; that could be due to a lot of different reasons. Maybe the supervising doctors are not very clear about what they are expecting the students to do, or the students do not really know what to do or how to do what they need to do to benefit from the clinical rotation. Sometimes, the doctors are not enthusiastic about teaching, or want to have students with them. This, in turn, disheartens the students… it may be useful to train the students about how to maximize their learning in the clinic. Setting personal learning goals is useful. For example, I had this goal in my head all along that even if I do not do much in a day, at least I need to take one history and do one physical examination…We really are not used to the physical load that clinics put on us…”  9F: “…sometimes it would be a challenging, during clinical rotations, to keep up with the studying, doctors’ expectations… At the same time, there is a lot of time that tends to be lost in transportation, trying to sleep early so I one can wake up early. It can get really messy…”  11F : “…one of the biggest challenges I had to face was communicating with my colleagues who had different viewpoints. A lot of times we were on different ends of the spectrum. I would spend my day worrying about what this person said to me without actually learning so that was kind of terrible…” |
|  | **Suboptimal attitude** | 2F: “…There were times where I felt ‘less than’, ‘doubtful’, ‘low’, or ‘burnt-out’…”  3M: “…as a consequence to COVID-19 and missing out on the clinical learning experience, the question that kept coming to my mind was: what if I am not capable and/ or confident enough to take care of patients because we have missed a lot of clinical learning in this year…”  4F: “… However, when it comes to our practical skills, I understand it was going to diminish…”  5F: “…at the beginning of the clerkship, I had a lot of self-doubt. I was not sure if I knew enough and if what I knew was correct because things keep changing. I was full of self-doubts; my first rotation was surgery at Mediclinic City Hospital…”  9F: “…I had a lot of self-doubt, hesitation, it was like testing the waters, scared and all that…my confidence did taper down in the past few months, with the quarantine and so, it took a toll on me when everything went online. I do not think my coping mechanisms kept up with that change…”  11F: “…I always have this feeling of inadequacy, in general. You just feel that you are not good enough, whether you are a consultant or a student…” |
| **Adaption** | **Internal** | 1M: “…It was a new kind of experience that we had to adapt to. I learned a lot in the transition to phase III and to that I am so grateful…”  2F: “…I developed a ‘thicker skin’, and I became more aware of social cues and more empathetic. These complementary skills cannot be learned through lectures and books, only... matters were no longer ‘black-and-white’. I had to learn to become receptive to feedback, whether it is negative (i.e., criticism) or positive…to acknowledge the importance of feedback and to use it to grow. In other words, to have a positive mindset towards feedback…”  3M: “…just learning from the experiences of my peers… I am trying my best to see how I can make up for the missed clinical experience due to COVID-19, not only for my self also for patients’ sake…”  4F: “…Humor also helped me; I really like joking about my situation, and after going through the psychiatry module, I learnt that humor was a mature coping mechanism… Also, the insight of when to rest and when to take breaks and that it is okay to take breaks was another good coping mechanism for me… it helped to know that COVID-19 was not in anyone’s control. Also, nobody could have predicted it… I am willing to continuously work towards developing myself as a doctor for the rest of my life…I have internalized that medicine as a practice is a work-in-progress, I am never going to be perfect…”  5F: “…I started my rotations at Mediclinic City Hospital doing surgery; I slowly found myself more drawn to the clinical aspect of things. I found myself staying at the hospital for a longer duration. Somehow my genuine interest and engagement was validating. My self-doubt was still there. Yet, I started to feel that I am doing okay, that I am doing something right. I loved what I was doing, I loved the hospital setting, the clinical environment…sometimes you get a really strong criticism from a doctor and then you kind of doubt yourself. One of the coping mechanisms was that I could cry…”  9F: “…then later my confidence built-up, I got really confident to the point that I would talk to doctors, even if I said a wrong answer or if I have concern that my question could be considered ‘stupid’. I just would shoot my shot, and I think that was good…my capacity to effectively manage my time was built as I progressed across the rotations. Since I realized the studying needs and doctors’ expectations for each rotation are different, so I came-up with different schedules accordingly. I had to make sure that not only am I covering adequate cases, I am doing my readings and my own studying…”  11F: “…I realize, I would not be in the hospital for clinical learning experiences if I knew everything. That gave me the peace of mind that this is a learning process and actually I will be continuously learning. It is unlikely to be competent in anything when you are doing it for the first time…the only coping mechanism that worked for me was choosing to distance myself from people who did not make me feel so great and approach doctors instead. I do not know if it is a healthy coping mechanism but I got to a point where I would actually stay back at the hospital until midnight to work and it was a great learning experience…” |
|  | **External** | 1M: “…a lot of the students helped me especially given that we were doing different rotations at the same time. So, for example, I started with psychiatry and after that I had family medicine so I used to speak to the students who finished family medicine and they would give me so many tips and so many tips about where to go, how to do matters, how to study, what resources to use, and that honestly really really helped me so much with preparing for the exam, with the experience, as a whole, with studying…I am A ‘Television shows/movies’ person so honestly my go to, whenever I am stressed or something, is always to watch an episode of a show/ series or a movie, or maybe go out with my friends to refresh and then go back to studying…family medicine weekly sessions on Thursdays were very useful on many levels… they were really helpful not only for the rotation, but for the exams. The way these team-based learning sessions were structured helped in maximizing the learning…by the time the exam came, I did not have to study anything new, I was practically revising what I had acquired and properly integrated throughout the family medicine rotation…I sincerely appreciate the family medicine lead; a very nurturing human-being…also, she always used to assign to us useful readings…”  2F: “… specifically this year, I am really really grateful for our batch and how much we have helped each other…I always had the option to go back to my colleagues, they would reassure me, and be like: ‘it is not unexpected to feel this way in this particular rotation’ or they may give me heads-up: ‘in this scenario, expect to feel/ think this way’. They would shed light on study resources to enable me to find my own way; self-learning. I am grateful for the fact that I had guidance, and that we all helped each other. I think that that was a huge enabling factor for me this year…my first rotation was in internal medicine in Mediclinic Welcare Hospital. The doctors were proactive when it came to teaching. With one of the doctors, we had multiple sessions where we picked-up patient files, and discussed and reflected upon cases. Another doctor would show us, from her textbooks, rare presentations of say skin manifestation that we do not get to see in this part of the world… I really appreciate when doctors go the extra mile to teach us beyond what we see every day in the hospital…”  3M: “…our batch have been very co-operative and very helpful in sharing their experiences which helped us in managing our own expectations in the upcoming rotations…a number of physicians were really friendly; the way they talked to us was not authoritative… for example, some regularly shared their opinions or how their professional lives, after medical school, turned-out to be…hearing, from different people, what they went through widened my horizons, encouraged me to reflect more on what I want to do…during lockdown due to COVID-19, I enrolled in a few online courses…one of them was called ‘Mindshift’; it taught me a lot of different things that were not discussed in the resilience course…”  4F: “…our batch was giving, and there was a strong sense of togetherness when it came to every single rotation. So that really helped us because we could rely on each other even though practically each of us had to do the work alone… the institution, as a whole, was taking steps to make sure our education was still at par of what they had intended prior to the onset of the pandemic…”  5F: “…the self-doubt was still there. I am so thankful, though, that I had very good, nurturing doctors there at Mediclinic City Hospital. They taught us with so much compassion and empathy. This was enabling despite my self-doubt…so one thing that was helpful for me was the meeting with my academic advisor which helped me destress as I was able to have an open discussion in a very safe environment…”  6F: “…transition to Phase 3 was tough, but the good thing for me was that I had a little bit of previous experience in the clinic, as part of a course in Phase 2; I knew what to expect…the quarantine made time management easier for me; it gave us ample time to study at our own pace…something that I think would have been very helpful to us in the rotation is having a faculty member onsite (present with us, in person), who is well informed about the hospital staff and us, to resort to when needed then and there… someone who knows how the hospitalize runs, who knows the doctors, who knows our schedules, someone who is ‘in between’ to facilitate…”  7M: “…I want to suggest having students go through a ‘transition exam’ between phase 2 and phase 3 that covers the organ systems mainly. Just a pass/ fail exam. I do not want it to affect the students’ Grade Point Average (GPA) or anything like that. If the students pass this exam, it will reassure them that we are ready to start year 4/ phase 3. This will also give them the impression that they are all on the same page, and by preparing for and undergoing this exam, they will be revising all the information that they had acquired in the first two phases/ 3 years, they will not need to spend time in year four revising everything that they had learned in the previous three years…”  9F: “…with medical schools we have a hard time trying to fit in extracurriculars however, if we can somehow have these more fitted in our calendar (e.g, have trips or extracurricular activities, that would help us destress)…”  10M: “…I think it would be helpful if we are provided with some sort of checklist of things or skills that we need to acquire during our clerkship so that we can seek out these opportunities even more…”  11F “…one biggest method which actually worked for me was basically going out into nature. Where I live, there is a walking track. It takes all your stress away, so for me for example if I spend a full day at the hospital, indoors seeing patients, interacting with doctors, which can get stressful. The best way for me was to go outdoors and walk on the track alone, reflecting on how my day went and that alone I think had the biggest impact on me. Every day, I looked forward to my walk… it greatly helped me during my rotations…I will never forget the meditation exercise. It was something really innovative and even the 10-minute sessions really had a good impact on me for the week. Personally, I found the course really helpful….” |
| **Added Value** | **Space** | 1M: “…it was fun… our Thursdays were packed with radiology and theoretical sessions of the rotation we are doing, so when we had the resilience skills’ building course session, it was kind of like a break, which was also fun and informative...I think the session where we meditated was really nice. It was honestly the first time I tried this kind of thing. So, it was a new experience, it was nice, it was interesting, and it was something you that I took back home…I enjoyed the sessions where we had ‘to do something’ like the ones where we needed to engage in the conversation or maybe when we had to mindful eat the date or prepare our own gratitude jar…”  3M: “…it was kind of fun, different than the rest of the lectures we were supposed to attend…I benefitted from the reassurance integral to this learning experience… that you are okay, and it is okay to be this way…I feel I needed someone to tell me that it is okay…”  4F: “…I think the setting helped a lot…sitting on bean bags; I was part of the second group, my batchmates from the first group kept raving about the fact that we get to sit on bean bags and relax instead of just being on chairs in our lectures all day…”  5F: “…I think it would have been really good if we took some of the sessions outside the university especially the meditation sessions- it would be a change in the air…”  6F: “…I found the guided meditation and the visualization sessions before the exam really nice and useful…”  10M: “…it was good to take a break from all the clinical and scientific things that we were studying. It was a place you could go, and switch off and relax, you know, just unwind…”  11 F: “…meditation sessions in the park would be a really good change…” |
|  | **Substance** | 1M: “…I learned a couple of skills which are useful… the meditation and mindfulness exercises...” 3M: “…doing simple acts mindfully such as washing my hands or anything else that is really simple… I gained the capacity and willingness to really focus on those simple activities… for example, if I am washing my hands just before going into a surgery, or washing my hands before touching the patient to do a physical exam or so…the highlights were chewing the date mindfully, eating it really slowly. I think the breathing exercises, as well, where I learned how to relax my body. I think those were really nice…”  4F: “…It was so needed for us as medical students…for the future, raising our level of awareness about the content of this course has been so impactful; I am extremely grateful for having had this experience…it was really helpful to learn psychiatry while doing this course as well because you got to learn and apply coping mechanisms while acquiring relevant technical knowledge… acquiring coping mechanisms… Especially when it came to understanding our own thoughts and emotions…the first skill that I gained is mindfulness and practicing mindfulness every day. For me, it was not a specific exercise that I needed to do. It was more like the skill became a part of how I am living… I noticed that when I am talking to say family members, I would be concurrently engaging with my thoughts: ‘oh I need to get this and this and this done’, and this would limit the experience of engaging with my family members and properly engage with them…the second skill would be reflection…I could reflect not only on my intentions as to why I am doing what I am doing, but also how I can do it better…”  5F: “…I like the content of the session but I would have loved it if we could have had been taught specific skills like cognitive behavior therapy (CBT) or Eye Movement De-sensitization and Reprocessing (EMDR) to handle patients who have post-traumatic stress disorder…” |
| **Sustainability** | **Shift** | 2F: “…mindfulness sessions. It was so helpful… applying it to our lives has been so impactful…I am, of course, nowhere close to living mindfully, all the time. Yet, I now have more awareness. There is an inner voice in my head constantly reminding me to be mindful, to bring awareness to what I am doing, to do matters mindfully. Similar to what my colleagues highlighted, bringing awareness to the simplest of activities: say, brushing my teeth. …”  3M: “…one of the coping strategies that I usually use is to talk with my friends, or go out with them and discuss matters; I sometimes do that with friends who are not from the university so I get a different perspective as to the matters I may be dealing with or issues that I may have…the breathing exercises were helpful, and I do practice them. So, anytime that I feel that I am overwhelmed, I just take deep breaths, with the intention of relaxing myself, so that I can focus on the task-at-hand rather than feeling overly anxious…”  4F: “…exercising mindfulness, meditation, and positive affirmations; I have seen a change in my life due to these practices, and my batchmates, the ones I have spoken to, also agree with me as well so I would say it has been a really positive experience…”  6F: “…I started meditating and I find that very helpful, as well as just taking time for myself. I learned to prioritize myself at some point and to create a balance so that I can study. Before I sleep, I take 5 minutes to just reflect, take a deep breath, sometimes I do yoga so all of these really help me to sleep and rest. When I wake-up, it is a new day and that really helps…the mindfulness exercises were really helpful although it takes time to actually get used to them. I know some students found the exercises difficult, while others found them easier. I benefited from them…”  11F: “…for a lot of my friends it (the course) was something very new and they expressed that it completely changed their lives in terms of learning to cope much better…” |
|  | **Commitment** | 2F: “…I also picked-up the habit of journaling- it came in handy around the COVID-19 times…”  3M: “…I have tried some of the techniques, but I failed at committing… For example, I tried keeping a diary like two or three times, somehow over time I end-up dropping the ‘ritual’ and focusing on other things or even forgetting about it or where I put the diary…doing simple exercises mindfully…I am not sure if I am doing it well; I have a tendency to feel agitated, to get distracted…It is sometimes hard to commit to staying mindful throughout an activity irrespective of its level of difficulty… especially when things are moving at a fast pace…”  7M: “…the fact that most of us are saying that we should have the course throughout the year means that it has actually impacted a majority of us…”  9F: “…I really wish I took that course now instead of before because although you gain skills, you practice them. The problem is maintenance. I should have maintained certain skills that I had gained from the course. I feel like it is now, during the pandemic, that we are truly being tested…” |
|  | **Impact** | 4F: “…living more mindfully enables me to do things in the same amount of time but in a way more relaxed manner…I now have a lot more clarity about what I am doing and where I am going… regularly reflecting enabled me to look back at my strategies of how I am doing things and instead of thinking: ‘okay I just need to get this done’, I say to myself: ‘okay how can I do it better?’ and then because of that, I think I have become a lot more efficient than I used to be…”  5F: “…I particularly liked the exercise of the gratitude jar. We were asked to write things we are grateful for. I took this concept into one of my rotations, as sometimes you focus so much on the negative and you forget the positive things in your life. So, for example, for one specific rotation, I remember it was for internal medicine, I added to my jar, using the extra colored-papers that I kept from the session, all that I appreciated and was grateful for from the rotation. I consciously directed my attention away from the negative aspects of my rotation…” |
| **Opportunities for Improvement** | **Experientiality** | 1M: “…I favored the sessions which were more interactive so I would say maximize the interactions throughout the course…the more interaction I had in the session, the more I took from the session…”  2F: “…the more interactive the session was, the more engaging and the more impactful it was. So, make all the sessions interactive, let them be built in the format of a workshop and not a lecture. This would be better. What was special about the mindfulness sessions was that they felt more like workshops. There were bean bags, they were interactive activities, while attaining the goal of the lesson. So maybe sessions like the one concerning time management can become more workshop like…”  3M: “…I do not want to think of this course as just another lecture; I want it to be more workshop-like with more interactions… I think one of my coping mechanisms is to spend more time with my friends. Hence, I would rather actually spend more time with my friends rather than attend a lecture that tells me to spend more time with my friends… if I was engaging with the content, at the same time engaging with the people around me, I will succeed in achieving these two things…”  6F: “…I think we can maybe have a few sessions about specific issues that we might run into during our clinical placement (e.g, how to deal with the situation when a doctor is angry at something or when we have too much on our plate) …. Basically, for the last couple of years some of us have got used to just sitting and studying without much movement, maybe introducing some cardio sessions for our well-being would be helpful…”  11F: “…I think it would be nice to have an online platform where people can anonymously talk about their experience in general, like a setting where people can share stories for example of how patients made a change in their lives or like how they coped with challenging situations in the clinic. This I believe would make us feel less alone when its is a shared experience…” |
|  | **Configuration** | 3M: “…if such techniques were brought-up earlier in our medical education journey, there would have been noticeable changes… the earlier these coping mechanisms are instilled in us, the more impactful they would be…”  4F: “…I really want the content of this course to be available to students of years 1, 2, and 3 because I feel like if I had received this learning experience in the previous years it would have been extremely beneficial…it is more about raising their level of awareness early on. I do not think it is about causing noticeable change among them…it is to normalize feeling stressed or anxious as part of the medical education journey and shedding light on potential coping mechanisms…”  5F: “…….it would have been good to have the sessions spread throughout the year because I noticed I needed it more later during the year…”  6F: “…the point about making the course optional I think is important because although several students are benefiting from it, unfortunately, it is not for everyone… if you make it optional, you can cater to those who really need it…”  8M: “…may be make it optional because I honestly feel it's not for everyone…. it did not really solve anything for me, but it might work for some other people…I think maybe prolonging the course instead of making it only for half the semester, make it all though the semester…I would like to propose to make it a club rather than a half a semester course…maybe instead of it being a half a semester course, probably make this into a club where we can introduce movie nights that show motivational movies or movies that inspire us…”  11F: “…if it were to be optional, it would identify only those students who really who need it, then people who might not appreciate the course that much…definitely having the course throughout the year rather than just half the semester would be beneficial to see the actual effect… basically because we will be attending the hospital in Year 5 all year round, having the meditation sessions all year round would be a much better option…” |
